# A Causal Model for Surveys of Exploratory Listening and Music Appreciation

**DOI:** 10.3390/bs15050676

**Published:** 2025-05-14

**Authors:** Henk Jacobs, Marc Leman, Edith Van Dyck

**Affiliations:** Department of Musicology—IPEM, Ghent University, 9000 Ghent, Belgiumedith.vandyck@ugent.be (E.V.D.)

**Keywords:** explorative music listening, music appreciation, survey, causal model, DAG, SEM

## Abstract

This paper integrates concepts from neurobiology, marketing and musicology to propose a causal model of music appreciation and exploratory listening, using directed acyclic graphs (DAGs) and structural equation models (SEMs). The key concepts are music appreciation (measured on a scale from 1 to 10), evaluations, experiences and the qualities of musical features, which the listeners explore and describe from a first-person perspective. The qualities are understood in terms of a satisfaction or dissatisfaction rating of operational features. The development of the causal model is based on a reiterative methodology involving surveys. Applying the causal model to a large survey of 800 listeners reveals that listeners adopt a slightly different causal pathway for their appreciation of liked versus disliked music. When listeners dislike music, the source of their dissatisfaction is more consistently attributed to the perceived or missed musical qualities rather than to their personal experiences. The iterative methodology and causal modeling offer a foundation for further investigation and refinement in various listening contexts.

## 1. Introduction

This paper investigates exploratory listening within the context of music appreciation. Exploratory listening is characterized by an active, open-ended engagement with music, where the listener seeks to uncover new patterns and deeper meanings, and music appreciation refers to the extent to which music is valued or desired. Conceptualizing the intersection of these two concepts presents a significant challenge, especially in terms of its practical application in survey research. Which theory supports a conceptual model for surveys of musical exploration and appreciation? And what methodology could turn theory into a survey instrument?

Section 1 gives a background of the research in the field of explorative music listening and appreciation. In Section 2, we introduce the reiterative methodology adopted in this study, and we review the concepts from neurobiology, musicology and marketing needed for developing the conceptual and statistical causal model. In Section 3 and Section 4, we introduce the questionnaire and the *Klara Top100* survey which provided the data for fine-tuning the causal model. Section 5 and Section 6 cover the correlation analysis and the statistical analysis of this survey, using the causal model. The discussion follows in Section 7, with a general conclusion given in Section 8.

### 1.1. Frameworks for Exploring Music

Frameworks for exploring music from the viewpoint of listening draw upon a broad research tradition having its roots in *Gestalt* psychology, esthetics and phenomenology (e.g., Seashore, 1938; Révész, 1944; Meyer, 1956; Wellek, 1963; Berlyne, 1971; Broeckx, 1981). Scholars aimed to understand how music impacts listeners, why listeners are fascinated by music and how musical features are related to musical experiences and appreciation. This type of research has been adapted to the paradigms of cognitive science and neuroscience (Leman & Schneider, 1997; Bader, 2018; Schneider, 2018), whose perspectives have deepened our understanding from a deeper, causal point of view. In line with this general trend, music listening has been studied with a focus on esthetics (e.g., Brattico & Pearce, 2013), music preferences (e.g., Fricke et al., 2021) and music appreciation (e.g., Thompson et al., 2023), among others. New perspectives based on causal principles from prediction processing, embodiment and neurobiology have broadened the research scope (e.g., Huron, 2006; Leman, 2007; Levitin, 2011; Vuust et al., 2022; Zatorre, 2024), drawing attention to the idea that music listening is an active process, involving the exploration of musical features in the search for experiences.

In line with these developments, some frameworks have been worked out, offering an overview and perspective for research. For example, Schäfer et al. (2013) found that people listen to music to regulate arousal and mood, to achieve self-awareness and as an expression of social relatedness. Thompson et al. (2023) identified three key pillars for investigating music appreciation: the first involved perceiving and internalizing musical structure; the second focused on activating networks of personal significance, identity and autobiographical memory; and the third, termed “source sensitivity,” referred to recognizing and engaging with the origins and contexts of music-making, including the personal attributes of musicians and the sociopolitical, historical and cultural contexts of music production. Reybrouck et al. (2024) proposed a distinction between three phases of musical information gathering: (i) describing music from a third-person perspective, relying on an objective analysis of its acoustic features; (ii) identifying potential mediating factors between the stimuli and the listener’s responses; and (iii) capturing the continuous, real-time physiological and cognitive–affective responses of the listener. Other studies in music preference have focused on emotions, personality and relationships, and have characterized music in similar terms (e.g., Alaei et al., 2022). Overall, these frameworks suggest that listeners engage in various forms of exploration, which subsequently shape their appreciation of music.

### 1.2. Causation as Listening

We believe that various components of music listening can be conceptualized from the viewpoint of a causal model. In adopting a theory-based causal model, it is possible to develop focused empirical tools and interpret data in the supporting theoretical framework.

In this modeling context, causality is understood through a statistical lens, which interprets correlation as a directional flow between variables that represent distinct elements of exploration and appreciation. As Pearl et al. (2016) aptly describe it, *“Think of causation as a form of listening; X is a cause of Y if Y listens to X and decides its value in response to what it hears”*. It is noteworthy that they use the concept of listening in this analogy. Applied to our case, we might posit that explorations of musical patterns cause experiences, because listeners “listen” to musical patterns and their experiences have values based on “what they hear”. Seen through a statistical lens, we apply correlation analysis subject to a chain of units which generate outputs based on what they receive as an input, thus constraining the correlations according to an assumed structure of processing. Ultimately, this causal chain culminates in a music appreciation score, which is the output of the last processing unit in the chain. Music appreciation is thereby assumed to be the causal result of exploring music, with experiences and subsequent evaluations as inputs. Note that in this modeling approach, causality is a logical construct, while in understanding the principles of music listening, causality points to a physical interaction at the micro-level (Bunge, 2017).

### 1.3. Quantification of Explorations

Building on the above background, we differentiate between inside and outside explorations, based on whether the focus is primarily on the musical experiences of the listener, or on the music itself, as seen from the viewpoint of the listener.

*Inside explorations* focus on the experiential dimension, including emotional responses, feelings of immersion and embodiment, memory associations, personal narratives and evaluations of these experiences. In music psychology, these inside explorations have often been probed using ratings on Likert scales (e.g., from 1 to 5). Questions thereby specify which observable is rated, for example, whether the listener felt an urge to move along with the music, or whether the listener felt strong emotions.

In contrast, *outside explorations* focus on structural features like gestures, melodies, rhythms, timbres and thematic elements (e.g., the social stratification in Beethoven’s pastoral themes) or contextual observations, such as the social connections formed during a concert. These explorations focus on various aspects of the music. They may be informed by previous knowledge such as the historical and sociocultural background of the composer, familiarity with the style or the listener’s previous experiences.

We believe that it is crucial to recognize that the listener’s exploration of music, including the verbal description of that exploration, is inherently subjective—selected, filtered, interpreted and thus conveyed from a first-person perspective. The first-person perspective, rather than the third-person perspective, is likely the most essential for understanding music exploration behavior in the context of music appreciation.

However, studies of outside descriptions revealing the listener’s perceived structures and thematic elements in music are rare. Most studies relate subjective measures to an objective baseline, using descriptions of musical features from a third-person perspective. Clemente et al. (2022) conclude that participants of their study differed remarkably in the extent to which their liking was explained by musical structures expressing balance, contour, symmetry and complexity, suggesting that esthetic sensitivity is a personal, albeit stable, trait. In this context, a first-person perspective of music could be an added value, as it draws attention to the features that the listener deems relevant.

Kunst and Van den Bergh (1984) provide an early example of music description from a first-person perspective. Their work can be understood as a conceptualization of exploratory listening at specific moments in time (Kunst, 1978). Examples such as *“From here on, I paid attention to the melody in the high register of the flutes”*, *“At this point, I was struck by a persistent rhythm”* and *“The horns recalled a hunting scene”* would illustrate how listeners describe their explorations of musical patterns and topics.

Clearly, these descriptions pose challenges for research. To incorporate them into a conceptual/statistical model, it is pivotal to develop a solution that acknowledges the subjectivity of these explorations while addressing the complexities of quantifying the personalized nature, because one subject may point to melody, while the other subject to rhythm or a topic. In what follows, we develop a solution based on marketing theory.

## 2. Modeling Approach

**Figure 1:**
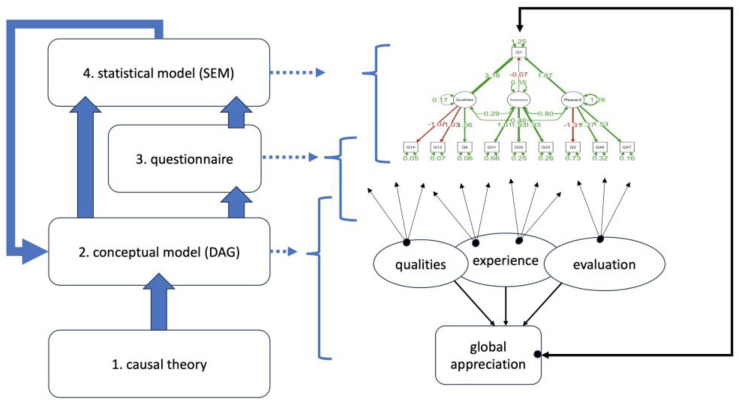
Scheme of the iterative modeling approach, involving a possible instantiation of a DAG and a SEM.

First, we introduce the overall modeling approach. It is based on different iterations in which theory and data analysis are mutually refined, as shown in Figure 1. The causal theory (1) offers insight in terms of the descriptions of mechanisms and dynamics. From that theory, we extract the conceptual model (2) (represented as a Directed Acyclic Graph, or DAG), the questionnaire (3) and the statistical model (4) (represented as a Structural Equation Model, or SEM). The left side of Figure 1 provides a schematic view of how these components are related, while the right side illustrates a DAG and an SEM. They appear as networks of concepts, with questions situated at arrow end points. The DAG and SEM are each other’s mirror, except that the SEM adds the computational tools for statistical analysis, defining the strengths of the connections among concepts. In the iterative process, the DAG, the questionnaire and the SEM are refined until a satisfactory result is obtained that still copes with the causal theory. This iteration uses data from a survey, with the questionnaire designed based on the theory itself.

**Figure 2:**
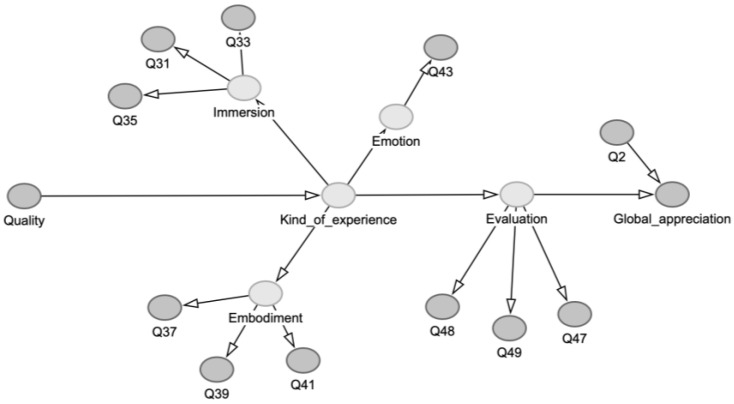
Directed Acyclic Graph (DAG) derived from the appreciation theory, using concepts and relationships between concepts. The horizontal arrows represent correlations (regressions) among variables, with an arrow indicating the direction from cause to effect. The non-horizontal arrows represent the confirmational factor analysis for defining variables, with arrows pointing to hidden variables and indicators.

In short, the data are analyzed through the SEM, which is derived from a conceptual model (DAG) based on the causal theories. The data serve to validate the conceptual model through the SEM, backed up by the causal theories. In this paper, Figure 2 shows a concrete DAG as a provisional final version of the iterative modeling approach.

The listener’s role is, first, to listen to a piece of music, and then to respond to questions about this listening activity. It is important to note that the context also plays a role in shaping the listener’s responses. In what follows, the context will be defined as listening to classical music radio, without further detailing the specific activities listeners engage in during listening.

### 2.1. The Causal Theory

We now turn our attention to the causal theory, using high-level concepts such as reward, pleasure, perceived value, immersion, embodiment and emotion to explain what it is about. To deal with outside observations and their quantification, we also include insights from marketing. These theories are mentioned here because they underpin the conceptual/statistical model.

First, we consider the viewpoint from neurobiology and musicology. *Neurobiology* explains music exploration–appreciation as a mild form of addiction, and its understanding is in terms of neurobiological processes (Berridge & Kringelbach, 2015; Panksepp & Bernatzky, 2002; Vuust et al., 2022; Zatorre, 2024). Accordingly, gratification (reward) is seen as an outcome from the brain’s chemistry, particularly involving neurotransmitters like dopamine and others. These chemicals diffuse throughout the brain, eliciting pleasurable sensations and triggering a desire for more—similar to a behavioral pattern of seeking stimuli that induce this reward, akin to addiction (Robbins & Clark, 2015). The scheme applies to food, sex, drugs, gambling, internet use and music, but the latter is generally considered to be mild and pro-social, compared to addiction that involves a loss of control, continued use despite negative consequences and neglecting one’s responsibilities or relationships. We then assume that some of these neurobiological processes penetrate consciousness, allowing listeners to observe, reflect and evaluate upon listening experiences in terms of their appreciation. For example, a dopamine release due to a reward can be experienced as pleasurable, then acknowledged and evaluated as such upon reflection (Gebauer et al., 2012; Zatorre & Salimpoor, 2013).

Musicology explains music exploration–appreciation with reference to musical qualities, thematic elements (cf. Hatten, 2003) and experiences of immersion, embodiment, anticipation, emotion and expression (Thompson et al., 2023; Reybrouck et al., 2024). Traditionally, the focus has been on perception-relevant pattern processing (e.g., tonal perception and rhythm perception (cf. Bader, 2018 for overviews)), while recent developments also focus on action, hence the role of prediction (Huron, 2006) and embodiment (Leman, 2007). In short, the contribution from musicology is about how listeners may explore and experience music. We assume that listening experiences involve emotional arousal, deep immersion, a physical urge to move or a profound emotional impact, and that listeners can subsequently reflect on these experiences, evaluating their effects. Upon reflection, the listener may also identify specific qualities in the music that are perceived to have caused these pleasurable experiences.

Finally, *marketing* considers the music appreciation score as the perceived value of a product, expressing the behavioral tendency to seek new stimuli (*wanting*). The product value reflects the overall customer satisfaction with the functionalities and qualities of the product. We believe that statements regarding music appreciation can be considered as examples of product binding, with the listener acting as a consumer and the piece of music being a product. In that scenario, the listener judges a product and gives it a high appreciation value when the music scores highly on features that are deemed important. Accordingly, the musical product can be judged in terms of satisfaction–dissatisfaction.

The product features can be assessed through questionnaires designed to probe customer satisfaction, delight and/or dissatisfaction and frustration. In the Kano model (Sauerwein et al., 1996; Mikulić & Prebežac, 2011; Kermanshachi et al., 2022), the features are characterized as *technical*, such as genre style, period, performer, tonal key and time signature characterizations; *operational*, such as a “cheerful flute”, “virtuoso oboe deployment” or “mystical sounds” calling for attention and bodily arousal; or *functional*, when arousal generates goosebumps, mood change or the feeling of being united with the music. Global appreciation is then the extent to which the consumer judges the product features as being attractive. Typically, in marketing, a score between 1 and 10 is used as a measure of global appreciation. The better a product scores in terms of being a product that a consumer finds important, the more likely it is that the consumer really wants to consume that product.

The key contribution of marketing concerns the description of *operational* features, which, in our approach, are both based on descriptions of features from a first-person perspective (using open questions), as well as descriptions of the degree of satisfaction or dissatisfaction of those features (using yes/no questions). In the Kano model, the consumer’s satisfaction–dissatisfaction is probed by “yes/no” questions about the features. Basically, consumers demand a certain quality of a product, and their satisfaction is higher when the quality of the expected product feature is higher. This is the so-called *one-dimensional* requirement for customer satisfaction. However, there are features which the consumer takes for granted, and if these are not fulfilled, there is low satisfaction or dissatisfaction. This is the *must-be* requirement for satisfaction. Additionally, if the product has features that are unexpectedly attractive (i.e., wow-features), then the satisfaction is high. This is the *attraction* requirement for satisfaction. Accordingly, the quality–satisfaction curve is not a straight linear curve, showing more satisfaction when quality is better. It is highly nonlinear, as the ends at both sides of the curve are bent: low satisfaction becomes much lower when certain features are taken for granted or are actually missing. High satisfaction becomes much higher when certain unexpected features turn out to be very attractive.

We focus on *operational* product features since *technical* product features provide information from a third-person perspective and *functional* product features are already addressed as descriptions of experiences using other questionnaires. Following the Kano model, we probe the satisfaction–dissatisfaction of operational product features using six questions that focus on whether features are loved, liked, indifferent, disliked, disturbing or missing. For example, to gauge whether a listener loves a piece of music, we ask: *“Does the piece of music have certain core qualities for you? These are characteristics that you immediately think of when you consider the piece of music. They give you a euphoric feeling, they touch you. They are also called wow-effects. Because of these core qualities, you love the piece of music. (yes/no)”*. This question relates to the attractive requirement and therefore, its score will be high. Another question is the following: *“It may be that the piece of music lacks certain elements that are important to you. Is this the case? (yes/no)”.* This question relates to the must-be requirement and therefore, its score will be low if the answer is positive (see Appendix A, questions Q6, Q8, Q10, Q12, Q14, Q16). A weighted sum of the responses to these six yes/no question results in a score that captures the satisfaction–dissatisfaction of outside explorations.

Of course, any comprehensive theory of music appreciation must also consider contextual influences. Factors such as the setting in which the music is heard (e.g., live concert vs. radio broadcast), the listener’s mood (e.g., happy, sad), demographic background (e.g., age) and environmental variables (e.g., cultural background) can significantly affect how listeners perceive and interpret their musical experiences. Therefore, when conducting surveys or assessments, it is key to account for these contextual factors to ensure the clarity and accuracy of participants’ responses, avoiding potential confusion or misinterpretation.

We also believe that music appreciation is independent of the specific type of music being listened to. Even though sad music may evoke a melancholic mood (Sachs et al., 2015), it can still contain qualities that trigger intense embodied experiences. These qualities and experiences can be highly valued by listeners, resulting in a high appreciation score. Conversely, happy music may not always receive a high appreciation score, especially if the listener feels that an essential component is missing. As a working hypothesis, we assume that, from a conceptual point of view, the underlying causality is the same.

To sum up, the causal theory holds that music can affect the listener’s reward, pleasure and desire system. This theory underpins a conceptual model of music appreciation focusing on explorations, experiences and evaluations. A key aspect of exploration concerns the identification of operational musical product features whose satisfaction/dissatisfaction can be quantified. The conceptual model also depends on various factors such as background, gender and musical context (radio, concert).

Overall, one could say that exploration and appreciation are based on elements from underlying physical and biological processing that penetrate the surface of consciousness. The conceptual model is about concepts and relationships on this surface, which can be probed using questions addressed to the listener (also called “indicators”).

### 2.2. The Conceptual Causal Model (DAG)

As an initial step towards data acquisition and modeling, we propose a causal model that outlines the variables and their interrelationships. This model is represented as a Directed Acyclic Graph (DAG), as illustrated in Figure 2.

In this DAG, the causal theory is reduced to a set of concepts, also called variables, each depicted by nodes, and the *causal* relationships among these concepts are shown as horizontal arrows. A cause–effect relationship between two concepts means that (i) there is a correlation or association between them; (ii) the flow of information is *directional* (indicated by the arrow), meaning that the effect is influenced by the cause; and (iii) *no confounding variables* can explain the observed association (cf. Bollen, 1989, chp. 3). The latter can be tested by examining the logic of causal relationships^1^.

In this DAG, Kind_of_experience represents the experiences caused by the explored musical qualities (Quality). These experiences lead to an evaluation, which ultimately informs Global_appreciation as the outcome. To use metaphorical terminology, Global_appreciation “listens to” Evaluation, which “listens to” Kind_of_experience, which “listens to” Quality. Additionally, Immersion, Embodiment and Emotion define Kind_of_experience. They are considered specific aspects of the experiences of an overall state of experience. Note that Quality directly reflects the outcome of the Kano model questions designed to assess the listener’s satisfaction with the explored musical patterns or topics that are deemed relevant to the type of experience being evaluated.

The connection between this DAG and the theory is that it assumes that explored musical qualities cause experiences in the listener, generating pleasurable feelings and a desire for more. The causal direction goes from qualities to experiences because the experiences logically require them, according to the causal theory known thus far. Accordingly, a wow-effect can be generated by certain features of the music, implying one causal direction, but not in the other direction.

The DAG also represents questions, each marked with the prefix Q. These questions have incoming arrows because the responses are generated by the latent variables. The latent concepts lie beneath the surface of what is observable, but we can probe them through questions, depicted here as gray ovals. This process is like fishing: we cannot see the fish beneath the water, but by casting our line (the questions), we catch what bites, and from those responses, we can infer insights about the hidden population below the surface.

The DAG presented above reflects the outcome of an effort to conceptualize the theory in view of statistical modeling. As for now, it represents an acceptable point in an iterative process of refinement (see Figure 1). The development of the DAG started from systems theory (cf. De Leeuw, 2000; Knowlton & Phillips, 2012), which offers a global picture of processing units and their causal relationships, defined in terms of inputs and outputs. Basically, systems theory posits that a subject is conditioning the states of the system, depending on variables such as age, gender, musical background, empathy and temperament, among others. As this subject explores musical features, the input goes from explored qualities to musical experiences, from experiences to evaluations and from evaluations to appreciation.

The next step in the conceptualization is inspired by the reward–pleasure–wanting scheme discussed by Leman (2016). In that scheme, information processing (i.e., prediction) is coupled with embodiment and social expression in a network that also connects to affect modules such as control, arousal and pro-social valence. Combining this conceptualization with the results from a previous survey in 2021 (see Section 4), we reason that three sub-categories of experiences (and associated questions) would be highly relevant for music appreciation: Immersion, Embodiment and Emotion. We thus obtain a rudimentary causal model that is further refined using tests with data from a survey. These tests involve statistical processing and model selection based on fitting criteria (see below). Any change in causal direction, adding new concepts that cluster questions as well as circular causality could be considered. For example, it would be possible to conceive a model in which musical quality is caused by evaluation.

In short, the conceptualization involves the setup of a causal model and an associated set of questions. Answers to these questions, via a survey, offer an opportunity for fine-tuning the causal model, because the structure underlying the questions offers room for re-arrangement. At this stage of the research, we find that a rationalized approach for model selection sketched above offers more solid ground for investigation, compared to a mere trial and error approach.

### 2.3. The Statistical Causal Model (SEM)

In this section, we convert the DAG into a Structural Equation Model (SEM). Mathematically, an SEM seeks to fit the covariance matrix Σ(θ) derived from the DAG (which includes concepts, relationships and answers to questions) with a covariance matrix Σ obtained from the data (which includes only the answers to the questions) (Bollen, 1989; Rosseel, 2012). The optimal fitting occurs when Σ = Σ(θ), where θ represents the parameters of the DAG. These parameters can be interpreted as the weight, or the relative importance, of the concepts.

The survey leads to 20 data variables which, after pruning (outlined below), result in a final set of 12 usable variables, based on the answers to questions by approximately 800 respondents. This results in a 12 × 12 covariance matrix (Σ), and a corresponding 12 × 12 covariance matrix embedded within a model defined by the parameters of the DAG Σ(θ). We then optimize Σ(θ) to approximate Σ. If the optimization is successful and meets the established criteria from the literature, we gain insights into the data from the perspective of the causal model.

In short, rather than treating the data as a collection of variables, our model suggests that the data follow a structured, causal logic as outlined by the DAG. Since the data are based on measurements, the purpose of the modeling is to test whether the model is supported by the data. However, the DAG’s validation does not guarantee that the underlying causal model is correct. Indeed, unlike falsification, which would directly disprove the model, validation only provides some degree of credibility to it. Additional support such as insights from neuroscience, musicology and marketing are needed to further substantiate the validity of the conceptual/statistical model.

The DAG described above can be transformed into an SEM using the following formulation:
model_1 <- ‘ Evaluation =~ Q47 + Q48 + Q49 Immersion =~ Q33 + Q35 + Q31 Embodiment =~ Q37 + Q39 + Q41 Emotion =~ Q43 Kind_of_experience =~ Immersion + Embodiment + Emotion Kind_of_experience ~ Quality Evaluation ~ Kind_of_experience Q1 ~ Evaluation + Q2 ### Q1 = Global_appreciation’

The description follows the syntax of the R package *lavaan* (Rosseel, 2012). The model specification is stored in a variable called model_1. The operator = ~ is used to define the confirmatory factor analysis (CFA) to create a new latent variable. For example, Evaluation is a new latent variable whose values are estimated from questions Q47, Q48 and Q49. The questions are listed in Appendix A. Similarly, Immersion, Embodiment and Emotion are new latent variables that together form the new latent variable Kind_of_experience. The operator ~ is used to define a regression analysis among existing (latent) variables, that is, after being created by the CFA. For instance, Quality is linked to Kind_of_experience, and Kind_of_experience is linked to Evaluation. Additionally, both Evaluation and Q2 are linked to Q1, which represents Global_appreciation. Q2 is a question about connection (*“With this piece of music I feel no connection”*).

The description implements the DAG within an SEM framework. The SEM can be analyzed from two perspectives: data analysis and data prediction. In the data analysis perspective, the goal is to estimate the weights of the latent concepts based on the observed data. From the data prediction perspective, the weighted concepts are used to predict the observed data. This could involve predicting the training data (retrodiction) or predicting new data (counterfactual prediction). In essence, the chain from answers to questions to appreciation can be reversed and interpreted as a predictive model for answering questions.

## 3. Selection of Questions

A complete list of all questions, excluding the open-ended ones from the original questionnaire (which are not addressed in this paper), can be found in Appendix A. Below, we briefly discuss the structure and processing of a selection of questions as specified in the structure of model_1. These are questions that have been retained after a correlation analysis (Section 5), see also Figure 2:

- Global_appreciation is estimated using a single question (Q1), scored on a scale from 1 to 10. This question reflects how eager the listener would be to listen to the music again.
- Quality is measured using six yes/no questions (Q6, Q8, Q10, Q12, Q14, Q16, detailed in Appendix A) that explore various aspects of satisfaction with musical qualities. It is then represented by a single score, which is a kind of weighted combination of the binary responses. The score is calculated as follows: for each answered question, “1” is assigned, while unanswered questions are marked with “0”. The weighted sum, called Quality, is then computed using the following formula:
  $$Quality=\frac{\left(Q6\times 4)+(Q8\times 1)+(Q10\times 0)+(Q12\times -1)+(Q14\times -4\right)}{Number\;of\;answered\;questions\;(excluding\;Q16)}\;+\;(Q16\times -1.5)$$
  Note that if Q10 is answered, it also adds to the number of answered questions. For example, if both Q6 and Q8 are answered, the score is (4+1)/2. If Q6, Q8 and Q10 are answered, the score is (4 + 1)/3. If Q12, Q14 and Q16 are answered, the score is −4. If Q14 and Q16 are answered, it is −5.5.
- Evaluation is based on three questions (Q47, Q48, Q49), assessing the personal value of the experience described in Kind_of_experience.
- Kind_of_experience is subdivided into three components: Immersion (Q31, Q33, Q35), Embodiment (Q37, Q39, Q41) and Emotion (Q45). These questions explore the type of experience generated by the music.
- Q2 stands as a separate question about connection (*“With this piece of music I feel no connection”*).

The questionnaire also includes demographic questions, such as hours spent listening to music (Q50), hours spent playing music (Q51), education level (Q52), age (Q53) and gender (Q54). Additionally, there are two questions regarding the arousal (Q3) and valence (Q4) attributes of the music. These questions categorize the music into four emotional states: angry (high arousal, low valence), sad (low arousal, low valence), happy (high arousal, high valence) and relaxed (low arousal, high valence) (cf. Russell, 1980; Eerola & Vuoskoski, 2012). These questions do not assess satisfaction or evaluation but instead reveal how the music fits into these emotional categories.

By utilizing this questionnaire, researchers can effectively gather data to better understand and analyze the components of music appreciation as described in the model.

## 4. Survey and Data

A version of the theory, the DAG and the SEM were initially developed and tested on data from a first survey conducted in 2021 at the Ghent Opera house. In this survey, listeners responded to the aria *Liebestod* from Richard Wagner’s opera *Tristan und Isolde*. After analyzing the data and refining the causal model, a follow-up survey was conducted in 2023 in collaboration with *VRT*-*Klara*, the classic music radio of the Flemish public broadcasting service. A major change in the 2023 survey, compared to the 2021 survey, was the incorporation of questions that probed participants’ outside observations using the Kano model to access listener satisfaction about explored musical patterns and thematic elements. In addition, the 2021 survey revealed consistent responses for questions probing Immersion compared to questions probing Emotion. Rather than putting all of these questions into one box, the questions were clustered in sub-categories of Immersion, Embodiment and Emotion, as explained above.

### 4.1. Context of the Survey

During the 2023 edition of the *Klara Top 100*, listeners were invited to submit their three favorite pieces of music. *Klara* then compiled a ranked list based on the number of submissions for each piece. Participants in the 2023 survey were given the opportunity to indicate their interest in participating in a follow-up survey from Ghent University. Those who expressed interest were directed to a survey platform (Qualtrics) hosted by Ghent University. A total of approximately 1250 listeners participated in the survey, with 807 completing the entire questionnaire. The data used in these analyses came from these 807 participants.

### 4.2. Questionnaire

The questionnaire was divided into two parts. In the first part, listeners were asked to focus on music they highly appreciated, such as their most preferred piece from the *Klara Top 100* survey. In the second part, they were asked to consider the music they would likely rate poorly (in Q57, the first part is coded as “*Liking*”, while the second part is coded as “*Disliking*”). Overall, the questionnaire contained 66 questions, including demographic and open-ended questions. The two parts (*Liking* and *Disliking*) used the same set of questions and the order of the two parts was counterbalanced across participants.

### 4.3. Participants

An overview of the participants reveals some notable trends. About 43% of participants are aged between 61 and 70 years, while only 17% are under 50. There are approximately twice as many females as male. Most participants report having a low level of formal music education. Their primary interaction with music is through listening, rather than playing. Specifically, 55% of participants listen to music for more than two hours a day, and 65% indicate that they do not engage in music-making themselves.

### 4.4. Distribution of Global_Appreciation

Global_appreciation (labeled as Q1 in the dataset), measured on a scale from 1 to 10, is categorized into *Liking* and *Disliking*. The most highly appreciated music receives a mean of 9.42, with a standard deviation of 0.88. Conversely, the least appreciated music scores a mean of 2.89, with a standard deviation of 1.66. Interestingly, the highest rated music tends to be consistently defined, while the lowest rated music shows greater variation. In some cases, the least appreciated music still receives an overall score of 5/10, and in rare instances, even 6/10.

## 5. Correlation Analysis

**Figure 3:**
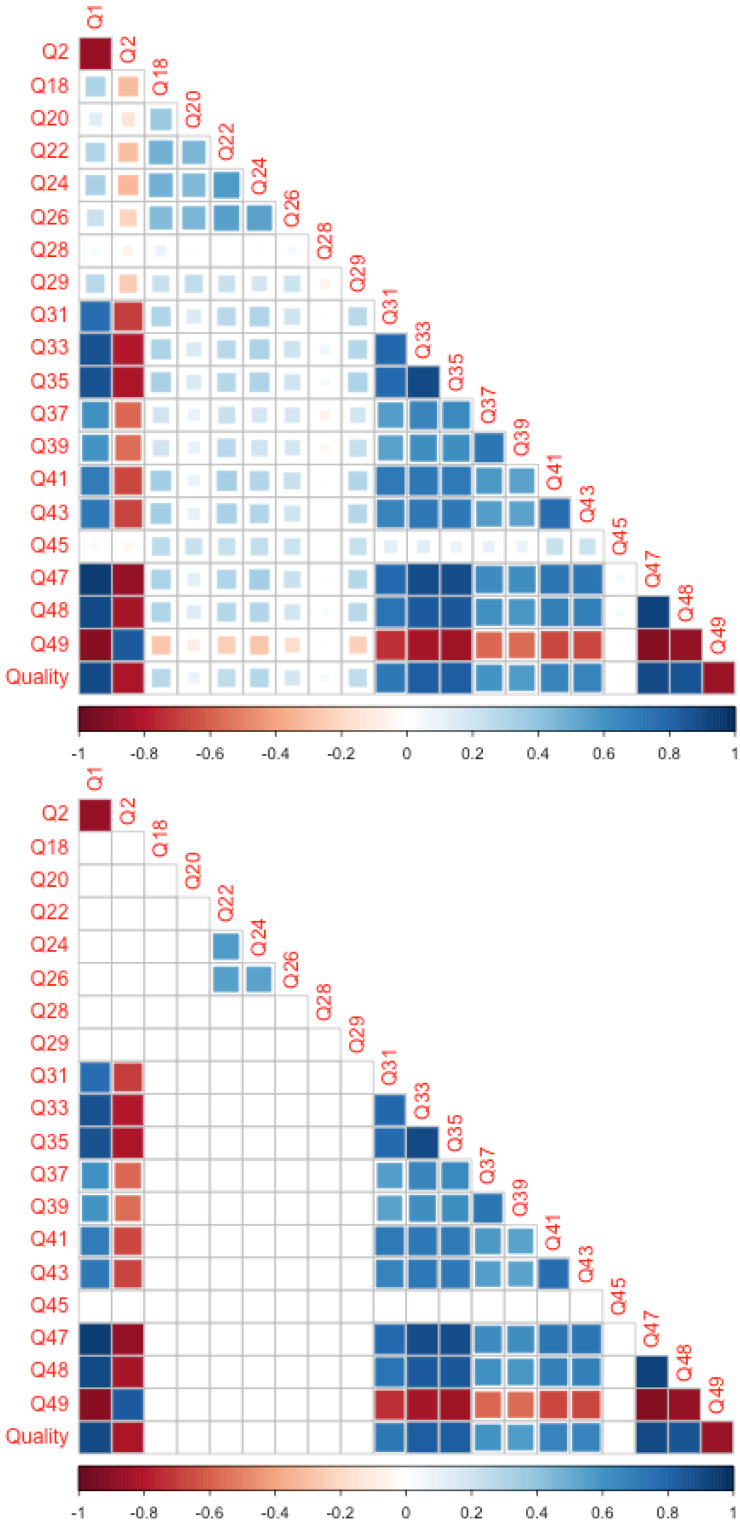
Original (**top**) and pruned (**bottom**) correlation matrices. The size of the square, as well as the color, reflect the correlation (range: −1, +1).

The questions to be retained for the causal model were selected based on their correlation with Global_appreciation (Q1). The rationale for pruning was that questions with a low correlation with Q1 were unlikely to significantly influence Q1, so they could be removed from further analysis. Figure 3 shows the correlation between all numerical questions.

To assess the contribution of each question to Q1, it was sufficient to examine the first vertical column (labeled Q1). Important questions will have high correlation values with Q1. We decided to remove questions with a correlation value below 0.5, or set them to zero.

The remaining questions are shown in the right panel: Q2, Q31, Q33, Q35, Q37, Q39, Q41, Q43, Q47, Q48, Q49 and Quality. Questions that explored the structural features of the music tended to show lower correlations. This might have been due to their complexity and/or the fact that listeners focused on subjective, selected aspects of the music. Additionally, Q45 was excluded, likely because listeners interpreted this question in different ways.

In summary, a streamlined version of the questionnaire has been created. The questions that meet the correlation threshold are now candidates for inclusion in the causal model.

## 6. Statistical Analysis Using SEM

In the following sections, we fit the SEM to our data. The goal of this process is to determine whether there is a structure that can explain or predict our data. The *lavaan* expression for fitting is:
semfit1 <- lavaan::sem(model_1, data = Data, group = “Q57”, meanstructure = TRUE)

Although the R package *lavaan* offers a variety of advanced features, we focus here on a simple fitting approach. We use Q57 to split the dataset into two groups: *Liking* and *Disliking*. The reason for splitting the dataset into *Liking* and *Disliking* is to test whether the models fitted to the two groups differ significantly, thereby examining whether concepts have different roles in the causal model.

### 6.1. Measurement Invariance

When fitting the models, we obtain parameter weights that describe the relationships between variables. However, before proceeding, we first assess whether the fitted model is acceptable by examining the discrepancy between Σ and Σ(θ). To do this, we use a technique called *measurement invariance*, which tests the consistency of a model across different groups and conditions. This is performed by comparing several nested models, where each successive model imposes increasingly restrictive constraints on the parameters. If measurement invariance is not established, differences in observed scores between groups may result from measurement bias rather than true differences in the underlying construct. Below, we provide a summary of the measurement invariance test, which is based on a hierarchy of nested models. Nesting refers to a situation where one model is a simpler, more constrained version of another, more complex model. This concept is crucial for comparing models to determine which one best fits the data:
Base Model: semfit1 <- lavaan::sem(model_1, data = Data, group = “Q57”, meanstructure = TRUE) Nested Model 1: semfit2<- lavaan::sem(model_1, data = Data, group = “Q57”, meanstructure = TRUE, group.equal = c(“loadings”)) Nested Model 2: semfit3<- lavaan::sem(model_1, data = Data, group = “Q57”, meanstructure = TRUE, group.equal = c(“loadings”, “intercepts”))

In the base model, model_1 is fitted to the data, with grouping based on the variable Q57. The meanstructure = TRUE argument indicates that the means are modeled. The nested model 1 includes an additional constraint: group.equal = c(“loadings”), which specifies that the factor loadings are equal across groups defined by Q57. The nested model 2 introduces an additional constraint group.equal = c(“loadings”, “intercepts”), which ensures that both factor loadings and intercepts are equal across the groups defined by Q57.

Nested models can be compared using likelihood ratio tests to assess whether the additional constraints significantly degrade the model fit. This hierarchical approach allows us to examine whether the differences in model parameters (such as loadings and intercepts) across groups are statistically significant. By comparing these nested models, we can make informed decisions regarding the invariance of model parameters across different groups, which is essential for validating the structural model across diverse populations.

The results are presented below (crosses indicate the best value over three outcomes):
####################### Model Fit Indices ########################### chisq df pvalue rmsea cfi tli srmr aic bic semfit1 902.093† 122 0.000 0.089 0.849† 0.810 0.083† 40633.042† 41042.366 semfit2 956.857 130 0.000 0.089† 0.840 0.811† 0.085 40671.807 41038.044† semfit3 1125.185 136 0.000 0.095 0.809 0.783 0.093 40828.134 41162.057

The following trends can be observed from the fit indices:

￭ Chi-square: All models show significant Chi-square values (*p* < 0.05), indicating that the models do not perfectly fit the data. This is common in large sample sizes, where even minor deviations from the model can lead to significant Chi-square results.
￭ RMSEA: All models have RMSEA values between 0.089 and 0.095, which fall within an acceptable range (typically below 0.08 for a good fit), suggesting a reasonable fit overall.
￭ CFI and TLI: CFI values range from 0.81 to 0.85, and TLI values range from 0.79 to 0.81, indicating a reasonable fit.
￭ SRMR: The model *semfit1* has the lowest SRMR (0.083), indicating the best fit in terms of standardized root mean square residual.
￭ AIC and BIC: The model *semfit1* has the lowest AIC value, suggesting it is the most parsimonious model among the three.

Based on these results, *semfit1* provides the best overall fit to the data. Given the minor differences between the models, we can reasonably assume measurement invariance. However, the fit indices also suggest that there may be areas where the models do not fully capture the relationships in the data. Further refinement of the model or exploration of alternative models might be necessary to improve the fit. Nevertheless, *semfit1* represents the best solution we have at this stage. Let us now take a closer look at the parameters.

### 6.2. Analysis of Parameters

In this section, we examine the estimated parameters for the latent variables and the regression model using the following command:
summary(**semfit1**, standardized = TRUE, rsquare = TRUE, ci = T)

The outcome of the summary is shown in Appendix B. In what follows, we use the *Std.all* values (last column) as our main entry point for discussion. *Std.all* provides estimates on a standardized scale (mean = 0, standard deviation = 1) and represents how much a standard deviation change in the latent factor (or regressor) is associated with a standard deviation change in the observed variable. We focus on these values in our discussion. Values close to 1 or −1 indicate strong relationships, while values close to 0 suggest weak relationships.

### 6.3. Analysis of Latent Variables

All latent variables fit well, except for Q2, which has a significant negative influence on Q1 in the *Disliking* group but not in the *Liking* group. Overall, the latent variables—Evaluation, Immersion, Embodiment, Emotion and Kind_of_experience—are well defined in both groups, with significant loadings on their respective indicators.

Next, we examine the contributions to Kind_of_experience for both groups:
Immersion:
Liking: Std.all = 0.870
Disliking: Std.all = 0.940Embodiment:
Liking: Std.all = 0.393
Disliking: Std.all = 0.694Emotion:
Liking: Std.all = 0.589
Disliking: Std.all = 0.299

The standardized loading for Immersion and Embodiment is higher in the *Disliking* group, while for Emotion it is higher in the *Liking* group. Overall, in the *Liking* group, we have determined Immersion as being the most important contributor, then Emotion and then Embodiment. Overall, in the *Disliking* group, we have determined Immersion as the most important contributor, then Embodiment and then Emotion. This presents an interesting contrast in the roles of Embodiment and Emotion across both groups. Embodiment plays a more significant role in the *Liking* group, while Emotion takes precedence in the *Disliking* group. To further interpret these findings, we now turn our attention to the regression results.

### 6.4. Regression Analysis

Overall, the model fit indicates that the responses can be predicted by the specified predictors, except for Q2 in the *Liking* group, which is not statistically significant.
Evaluation ~ Kind_of_experience
Liking: Std.all = 0.597
Disliking: Std.all = 0.857Kind_of_experience ~ Quality
Liking: Std.all = 0.141
Disliking: Std.all = 0.453Q1 ~ Evaluation
Liking: Std.all = 0.497
Disliking: Std.all = 0.523Q1 ~ Q2
Liking: Std.all = −0.010 (not significant)
Disliking: Std.all = −0.098 (significant)

Overall, the standardized regression coefficient is notably higher in the *Disliking* group, suggesting a stronger relationship than in the *Liking* group.

The role of Quality is particularly interesting. In the *Disliking* group, Quality contributes strongly, while in the *Liking* group, it contributes weakly. Typically, in the *Disliking* group, Quality will have a low (negative) value, strongly influencing the value of Kind_of_experience, while in the *Liking* group, Quality will have a high (positive) value weakly influencing the value of Kind_of_experience.

In this causal network, Quality is interacting directly with Kind_of_experience, while the interaction with Immersion, Embodiment and Emotion is only indirect. The group effect defines how strongly Kind_of_experience, defined by Immersion, Embodiment and Emotion, “listens” to an external source. In the *Disliking* group, it strongly “listens” to this external source, while in the *Liking* group, it weakly “listens” to this external source. Accordingly, in the latter, the defining variables of Kind_of_experience will define its role in the rest of the causal chain, rather than Quality. The indirect interaction thus means that if the external source is stronger or weaker, the defining variables become less or more important.

In summary, the causal model suggests that listeners in the *Liking* group tend to neglect an external source, while in the *Disliking* group they tend to rely on an external source, here identified as their satisfaction with musical features (Quality). Moreover, as explained in the analysis of latent variables, the definition of Kind_of_experience is different for *Liking* and *Disliking*, parallel to the weak or strong contribution of Quality.

### 6.5. Testing Against Other Models

While validation lends credibility to the theory, one might question whether the test model (model_1) is truly the best option. What if a model were proposed where Quality and Kind_of_Experience have a direct link to evaluation? Below, we present the syntax for model_2 along with its model fit indices.
model_2 <- ‘ Evaluation =~ Q47 + Q48 + Q49 Immersion =~ Q33 + Q35 + Q31 Embodiment =~ Q37 + Q39 + Q41 Emotion =~ Q43 Kind_of_experience =~ Immersion + Embodiment + Emotion Evaluation ~ Kind_of_experience + Quality Q1 ~ Evaluation + Q2’

In this model description, Quality is no longer treated as cause of Kind_of_experience. Rather, it is added, together with Kind_of_experience, causing a change in Evaluation.
####################### Model Fit Indices ########################### chisq df pvalue rmsea cfi tli srmr aic bic semfit1_model_2 988.969† 122 0.000 0.094 0.832† 0.788 0.098† 40579.822† 40988.863 semfit2_model_2 1042.164 130 0.000 0.093† 0.823 0.791† 0.101 40617.017 40983.002† semfit3_model_2 1209.020 136 0.000 0.099 0.792 0.765 0.105 40771.873 41105.564

A comparison of the model fit indices of model_2 and model_1 reveals that model_1 still provides the best combination of fit indices, with the lowest AIC and BIC values across all models tested. This suggests that model_1, where Quality is considered as a causal factor for Kind_of_experience, offers a better fit than model_2, in which Quality does not have a direct causal impact on Kind_of_experience (model_2).

## 7. Discussion

### 7.1. Statistical Modeling

Statistical modeling shows that when a listener likes the music, Quality, whose origin refers to exploratory listening quantified in terms of consumer satisfaction/dissatisfaction, tends to have less influence on the overall network leading to global appreciation, compared to when the listener dislikes the music. Obviously, we must consider this weight as part of a larger network of components and relationships. The lower weight in the *Liking* group can be explained by the fact that listeners may recognize varying qualities in the music, while their experiences remain more consistent. In contrast, when listeners dislike the music, the source of their dissatisfaction is more consistently attributed to the perceived or missed musical qualities, rather than to their personal experiences. For example, liked music does not necessarily have to contain a wow-effect, while the disliked music is often missing something. This translates to scoring that is more consistent for the *Disliking* group.

In contrast to the initial hypothesis that music appreciation is independent of the specific type of music being listened to, this finding reveals subtle differences in the causal pathways determining the perception of music liked and disliked by the listeners. Overall, when listeners dislike the music, they more consistently attribute their dissatisfaction to the quality of the music. In contrast, when listeners like the music, they attribute their enjoyment more to the overall experience rather than focusing on the quality alone.

### 7.2. Modeling Exploratory Listening Behavior

Exploratory listening behavior has been quantified based on satisfaction with the explored features, although the specific features were not identified here. In the survey, however, listeners were able to provide examples of the types of features they had in mind through open-ended questions. While further analysis of these open responses is needed to better understand the nature of these explorations, our findings demonstrate that a quantified assessment of exploratory listening behavior—based on satisfaction with musical features and topics—is both possible and relevant as a component in statistical modeling. Moreover, testing the causal influence of Quality suggests a model where Quality plays a central role in shaping experience. Refining the application of the Kano model to listening behavior could further enhance the causal role of Quality in the model.

### 7.3. Experiences

Interestingly, Immersion emerges as the most consistently important predictor of experience, while Embodiment and Emotion play different roles depending on whether music is liked or disliked. When the music is liked, Emotion is slightly more prominent than Embodiment, whereas when the music is disliked, Embodiment becomes more prominent than Emotion. Despite this nuance, the consistent importance of Immersion in both liked and disliked music may be somewhat surprising, given that the traditional music perception literature has largely focused on appraisals of beauty, enjoyment or emotional responses. The finding that Immersion, and the associated experiences of unity and absorption, is a stable predictor for appreciation is line with the findings of our first survey in 2011 (not published), as well as with the recent empirical research of Swarbrick et al. (2024) showing that absorption connects to experiences of feeling moved, in awe, connectedness and enjoyment. Ultimately, the emphasis on Immersion and Embodiment highlights broader questions about how humans engage with and inhabit their environments.

### 7.4. The Quality/Experience Problem

The relationship between Quality, Kind_of_experience and Evaluation is intricate, and this approach still overlooks the influence of context. It is possible that satisfaction or dissatisfaction with explored listening behavior can vary depending on the context. Listeners may feel satisfied with their listening experience in a concert setting but dissatisfied with their listening experience in a radio setting. For example, modernist works like Pierre Boulez’s *Pli selon pli*, an orchestral piece composed in 1957 and beyond, might require a concert environment where the attentive listeners can immerse themselves in the dynamic blending of timbres from different orchestra sections. Yet, when the same piece is broadcasted on the radio, it may become less enjoyable due to a lack of full attention or insufficient preparation. In this case, the listeners may attribute their low appreciation to the very same musical qualities that were admired in the concert setting but which they find dissatisfying given the radio broadcast context. The radio context simply does not support the piece, resulting in a diminished experience.

Interestingly, our dataset does not show any cases where high quality is associated with low experience or evaluation. Since participants were asked to provide examples of music they disliked when heard over the radio and then justify this dislike in terms of their satisfaction/dissatisfaction with the associated musical patterns or topics, they seem to have consistently rationalized from a first-person perspective, meaning that the musical features were considered truly operational for the experiences.

### 7.5. Limitations

It is important to acknowledge the limitations of our approach. Currently, our study is confined to a single context, that being radio listening. Expanding this to include multiple contexts could provide a more comprehensive understanding, particularly regarding how listeners may have contradictory music appreciations and exploratory listening, depending on the setting. Despite this limitation, we believe our methodology successfully demonstrates how to develop a practical tool for assessing music appreciation and exploratory listening. This tool can be applied across various contexts, including both radio and live concert settings.

A further point of discussion, perhaps, is how exploration and experiences are related. Here, we assume a direct causal line from exploration to experiences, but it is likely that past experiences have influenced explorations. After all, the idea that exploration is prediction-driven supports the idea that past experiences play a crucial role in explorations. Longitudinal aspects of exploration and appreciation have not been considered in the current study.

A further limitation concerns the validation and its associated model selection. As mentioned above, validation implies that the causal model, described as the conceptual model in the DAG of Figure 2 and as the statistical model in the SEM, might not be the final “true” model. In the future, it may turn out that alternative causal models score better. Their implementation may turn out to be subtle, depending on many factors.

Overall, we believe that the iterative methodology, which subscribes to Bayesian epistemology, is promising. Adopting this approach for surveys in different contexts could improve the causal modeling approach.

## 8. Conclusions

In this paper, we draw on concepts from neurobiology, marketing and musicology to develop a causal model of exploratory listening and music appreciation. The methodology emphasizes the need to validate the causal model through both a theory-informed questionnaire and theory-driven modeling.

Overall, the DAG-SEM modeling of data from the *Klara Top 100* (2023) survey complies with a causal theory of music appreciation, incorporating exploratory listening. Fundamentally, the theory focuses on the listeners’ reflection on their exploratory listening and the verbal articulation of that experience. The findings of this study, coupled with a theory-informed questionnaire, may provide a valuable tool for future surveys in this field.

## Data Availability

The original contributions presented in this study are included in the article. Further inquiries can be directed to the corresponding author.

## Note

1 See Pearl et al. (2016). The causal logic of a DAG can be tested using the tool Daggity. It involves evaluating whether the assumed causal structure is consistent, that is, whether certain sets of variables are conditionally independent given other sets of variables. Our DAG is safe. No adjustment is necessary to estimate the total effect of Kind_of_Experience on Global_Appreciation, meaning that there are no confounding paths between the variable Kind_of_Experience and the outcome variable Global_Appreciation.

## Appendix A

(#tab:chapListener_datasetQ) Questionnaire with labels, question summaries and questions. L5 means Likert scale from 1 to 5		
**Label**	**Question Summary**	**Question**
Title	Title Open	Give the title of the musical piece
Composer	Composer Open	Give the name of the composer
Q1	Global_appreciation 1 to 10	Give your global appreciation score
Q2	Connection L5	|With this piece of music I feel no connection
Q3	Arousal L5	|In this piece I hear density, energy, activity, excitement
Q4	Valence L5	|In this piece I hear joy, optimism, positive emotions
Q6	Loving Yes(1)/No(2)	|Does the piece of music have certain core qualities for you? These are characteristics that you immediately think of when you consider the piece of music. They give you a euphoric feeling, they touch you. They are also called wow-effects. Because of these core qualities, you love the piece of music.
Q8	Liking Yes/No	|Question 2: Besides core qualities, the piece of music may have distinctive elements for you that are certainly worth listening to. They give you a good feeling and often create a ‘click’ with the piece of music. Do you recognize these elements?
Q10	Indifferent Yes/No	|Question 3: Does the piece of music have certain distinctive elements for you that you don’t care for? These may be found, for example, in transitional passages. They have no positive, but also no negative effect on your musical experience.
Q12	Disliking Yes/No	|Question 4: Even if the piece is your first *KLARA top 100* piece, it may still have certain distinctive elements that you don’t like. Do you recognize this?
Q14	Disturbing Yes/No	|Question 5: Does the piece of music have certain distinctive elements that bother you or even annoy you?
Q16	Missing Yes/No	|Question 6: It may be that the piece of music lacks certain elements that are important to you. Is this the case?
Q18	Sphere L5	|Statement 1: The atmosphere I hear in the music influences my choice.
Q20	Structure L5	|Statement 2: The structure/composition of the piece of music influences my choice.
Q22	Melody L5	|Statement 3: The melody of this piece of music influences my choice.
Q24	Harmony L5	|Statement 4: The harmony/ensemble singing in this music influences my choice.
Q26	Time L5	|Statement 5: The rhythm and tempo of this piece of music influences my choice.
Q28	Texture Categorial	|Does the piece of music have vocals or is it instrumental?
Q29	Texture L5	|The lyrics influence my choice.
Q31	Attention L5	|Statement 1: Generally, this piece of music demands my full attention (for example, ‘forgetting time’, ‘full concentration on the music (and the lyrics)’, ‘completely shut off from the surroundings’).
Q33	Absorbtion L5	|Statement 2: Generally, it feels as if this piece of music and I are one (for example, ‘being completely captivated by the music’, ‘feeling one with the music’, ‘feeling after listening as if I have been on a journey’).
Q35	Engagement L5	|Statement 3: Generally, I feel completely engaged with this piece of music (for example, sympathy, empathy, and identification with the performer(s), composer, characters in the music, meaning of the music).
Q37	Moving L5	|Statement 4: Generally, I move automatically with this piece of music (for example, tapping to the beat, swaying, conducting along).
Q39	Participation L5	|Statement 5: Generally, I sing, whistle, or hum along with this piece of music.
Q41	Physical L5	|Statement 6: Generally, I experience physical sensations with this piece of music (for example, goosebumps, shivers down the spine, lump in the throat, tears of emotion, heart beats faster).
Q43	Emotions L5	|Statement 7: Generally, I experience certain emotions with this piece of music (for example, nostalgia, energy, happiness, sadness, inspiration).
Q45	Mood L5	|Statement 8: Generally, my mood changes when listening to this piece of music.
Q47	Enjoying L5	|I can intensely enjoy this piece of music.
Q48	Touching L5	|This piece of music affects me in a positive way.
Q49	Annoying L5	|I can get extremely annoyed by this piece of music.
Q50	Listening Categorial	|How long do you typically listen to music in a day?
Q51	Playing Categorial	|How long do you typically play music yourself in a day?
Q52	Level Categorial	|How would you rate your musical ability?
Q53	Age Categorial	|What is your age?
Q54	Gender Categorial	|You are

## Appendix B

Group 1 [Liking]: Latent Variables: Estimate Std.Err z-value P(>|z|) ci.lower ci.upper Std.lv Std.all Evaluation =~ Q47 1.000 1.000 1.000 0.315 0.674 Q48 0.848 0.114 7.422 0.000 0.624 1.072 0.267 0.383 Q49 −0.368 0.054 -6.756 0.000 −0.474 −0.261 −0.116 −0.337 Immersion =~ Q33 1.000 1.000 1.000 0.671 0.783 Q35 0.702 0.050 13.968 0.000 0.604 0.801 0.471 0.600 Q31 0.856 0.058 14.747 0.000 0.742 0.969 0.574 0.649 Embodiment =~ Q37 1.000 1.000 1.000 0.896 0.727 Q39 0.957 0.104 9.205 0.000 0.753 1.161 0.857 0.672 Q41 0.408 0.052 7.860 0.000 0.306 0.509 0.365 0.381 Emotion =~ Q43 1.000 1.000 1.000 0.681 1.000 Kind_of_experience =~ Immersion 1.000 1.000 1.000 0.870 0.870 Embodiment 0.602 0.094 6.412 0.000 0.418 0.786 0.393 0.393 Emotion 0.688 0.075 9.156 0.000 0.540 0.835 0.589 0.589 Regressions: Estimate Std.Err z-value P(>|z|) ci.lower ci.upper Std.lv Std.all Evaluation ~ Kind_of_exprnc 0.322 0.040 8.028 0.000 0.244 0.401 0.597 0.597 Kind_of_experience ~ Quality 0.089 0.027 3.300 0.001 0.036 0.142 0.152 0.141 Q1 ~ Evaluation 1.382 0.160 8.630 0.000 1.068 1.696 0.435 0.497 Q2 −0.015 0.046 −0.318 0.750 −0.105 0.075 −0.015 −0.010 Group 2 [Disliking]: Latent Variables: Estimate Std.Err z-value P(>|z|) ci.lower ci.upper Std.lv Std.all Evaluation =~ Q47 1.000 1.000 1.000 0.568 0.787 Q48 0.988 0.049 20.044 0.000 0.892 1.085 0.561 0.761 Q49 −0.742 0.067 −11.033 0.000 −0.874 −0.610 −0.421 −0.420 Immersion =~ Q33 1.000 1.000 1.000 0.617 0.837 Q35 1.027 0.040 25.803 0.000 0.949 1.105 0.633 0.854 Q31 0.775 0.059 13.041 0.000 0.659 0.892 0.478 0.467 Embodiment =~ Q37 1.000 1.000 1.000 0.766 0.901 Q39 0.904 0.044 20.712 0.000 0.818 0.989 0.692 0.800 Q41 0.413 0.059 6.989 0.000 0.298 0.529 0.317 0.261 Emotion =~ Q43 1.000 1.000 1.000 1.287 1.000 Kind_of_experience =~ Immersion 1.000 1.000 1.000 0.940 0.940 Embodiment 0.917 0.058 15.852 0.000 0.804 1.031 0.694 0.694 Emotion 0.664 0.086 7.766 0.000 0.497 0.832 0.299 0.299 Regressions: Estimate Std.Err z-value P(>|z|) ci.lower ci.upper Std.lv Std.all Evaluation ~ Kind_of_exprnc 0.840 0.051 16.573 0.000 0.740 0.939 0.857 0.857 Kind_of_experience ~ Quality 0.205 0.017 12.108 0.000 0.172 0.238 0.354 0.453 Q1 ~ Evaluation 1.517 0.109 13.914 0.000 1.303 1.731 0.861 0.523 Q2 −0.142 0.044 -3.190 0.001 −0.229 −0.055 −0.142 −0.098
